# Impact of audio on navigation strategies in children and adults with dyslexia

**DOI:** 10.1007/s11881-022-00271-3

**Published:** 2022-10-04

**Authors:** Carolien A. N. Knoop-van Campen, Eliane Segers, Ludo Verhoeven

**Affiliations:** 1 Behavioural Science Institute, Radboud University, Montessorilaan 3, 6525 HR Nijmegen, The Netherlands; 2 Department of Instructional Technology, University of Twente, Enschede, The Netherlands

**Keywords:** Audio-support, Dyslexia, Multimedia learning, Navigation strategies

## Abstract

Children and adults with dyslexia are often provided with audio-support, which reads the written text for the learner. The present study examined to what extent audio-support as a form of external regulation impacts navigation patterns in children and adults with and without dyslexia. We compared navigation patterns in multimedia lessons of learners with (36 children, 41 adults), and without dyslexia (46 children, 44 adults) in a text-condition vs. text-audio-condition. Log files were recorded to identify navigation patterns. Four patterns could be distinguished: linear reading (linear), linear reading with rereading (big peak), reading with going back to previous pages (small peaks), and a combination of strategies (combined peaks). Children generally used linear navigation strategies in both conditions, whereas adults mostly used combined-peaks strategies in the text-condition, but linear strategies in the text-audio-condition. No differences were found between learners with and without dyslexia. Audio-support does not impact navigation strategies in children but does seem to impact navigation strategies in adult learners, towards the use of more linear navigation patterns, reflecting less self-regulation.

Reading to learn is becoming more and more a task in which the learner is required to integrate information from multimedia while using different modalities. When going through a multimedia document, multimodal information (spoken and written text) must be integrated and combined to form a coherent mental model (Caccamise et al, 2015; Juvina & Van Oostendorp, 2008). Learners follow different navigation strategies to build such a mental model, as seen in the level of linearity with which they go through the material (Paans et al., 2020). Audio-support guides learners in a linear way through the material. It would require some form of self-regulation of the learner to ignore this guidance and follow their preferred navigation pattern. Especially for children, who have lower self-regulation skills (De Jong & van Joolingen, 1998), this may be a difficult task (Salmerón & García, 2011). Interestingly, learners with dyslexia are often provided with audio support to compensate for their reading problem (Ghesquière et al., 2010; Gregg et al., 2009). These learners often approach (multimedia) learning materials differently than their typically developing peers (e.g., Heiman & Precel, 2003; Polychroni et al., 2006). Adding narration to written text may thus impact the navigation strategies of learners with dyslexia differently than those of typically developing peers. We, therefore, examined to what extent adding audio-support to multimedia environments affected navigation strategies in children and adults with dyslexia compared to their typically developing peers.

## Multimedia learning 

Nowadays, children as well as adults are increasingly provided with multimedia, for example in instructional videos and via the internet. Educational textbooks contain both written text and pictures. In addition, learners are increasingly provided with audio-support: the opportunity to listen to the information in schoolbooks by means of audio-software or spoken materials (narration). Multimedia have thus become an integral part of educational learning environments.

The cognitive theory of multimedia learning (CTML; Mayer, 2005) provides a broad theoretical framework with various principles for creating efficient multimedia learning environments. While the theory focuses on learning outcomes, and not on learning processes, it is a framework that may be used to understand learning processes as well. The design principles in CTML focus on minimizing the burden on the working memory so that working memory capacity can be used to process the information stored in long-term memory. The CTML (Mayer, 2005) is based on three assumptions: the dual channel assumption — there are two separate channels for processing visual and verbal material (Paivio, 1986); the limited capacity assumption — only a limited amount of information can be processed in a channel at any one time (Baddeley, 1999); and the active processing assumption — meaningful learning occurs when relevant material is selected, organized, and integrated (Wittrock, 1989).

One of the design principles in CTML is the redundancy principle. It states that the simultaneous presentation of identical information in visual (written text) and oral (audio-support) format hampers the learning process (Mayer & Fiorella, 2014). As visual and audio channels have to process the same information, the unnecessary processing of the information twice requires additional working memory capacity, which is no longer available for learning (Mayer & Fiorella, 2014). By presenting both written words and pictures, the visual channel becomes overloaded (similar as in the modality principle), and double information (words in written and spoken form) must be processed. In addition, since learners are provided with multiple sources of information, which all must be integrated and combined to form a coherent mental model (Graesser, 2007), regulating one’s own learning process becomes more crucial (Azevedo & Cromley, 2004).

## Navigation in multimedia learning environments

To combine these multiple sources of information learners engage in different levels of self-regulation, as the ability to self-regulate during formal learning develops throughout childhood, young children are thought to be mostly incapable of regulating their learning (De Jong & van Joolingen, 1998). Throughout adolescence, self-regulation skills develop (De Jong & Van Joolingen, 1998; Zimmerman, 2000), and learners become increasingly better at regulating their learning proces.

Learning is not only dependent on the internal regulation of the learner (Pressley & Harris, 2006). The learning environment also provides external regulation via its design in which the material prompts the learner in a certain direction. Task features may thus affect the learning process (Greene & Azevedo, 2010; Winne & Hadwin, 1998). Especially audio-support can be considered a task feature that could affect students’ self-regulation as it provides direct steering within the task. As the audio voice may “pull” learners in a linear way through the learning environment, this may go at the expense of learners’ own choices (on what to read and in which order) and decreases the opportunities to use their self-regulation skills. Self-regulation activities such as making decisions about which parts of the lessons learners want to (re)visit and the motivation to be actively engaged could then decrease. Such external regulation may go at the expense of internal regulation.

A recent hypermedia study confirmed this line of thought and found that learners more often used a linear reading pattern in higher structured learning environments with more external regulation (Paans et al., 2020). Another study showed that learners’ approach of a task can be affected by task conditions (Pieschl et al., 2012). This fits with the statement that self-regulated learning is a “dynamic and developing process” (Boekaerts & Corno, 2005, *p*. 208). In addition, a recent study on multimedia and reading strategies in secondary school students showed that audio support had a negative effect on students’ reading comprehension strategy (Knoop-van Campen et al., 2022). In this study, we found that audio elicited less efficient reading behavior: students made less reader-initiated decisions but tend to follow the audio. We therefore argue that information processing must be active and that audio makes it less active. Thus, a learner may show less self-regulation (less reader-initiated decisions) when audio-support increases external regulation.

## Visualizing self-regulation in multimedia learning environments

An objective and non-intrusive way to visualize learners’ self-regulating in multimedia environments is by documenting the movement through such environments and showing learners navigational path based on log file data (Barab et al., 1996; Jeske et al., 2014; Lawless & Kulikowich, 1996). An example of a study using such log data to examine learners’ navigation activities in a (hyper)media setting used plotted graphs in which was shown when learners moved to a different page in the multimedia environment. Paans et al. (2020) showed that various navigation patterns could be distinguished, e.g., linear reading, selective reading, and unpredictable reading. These navigation patterns differed in terms of linearity with which students went through the online learning material.

## Audio-support and dyslexia

Visualizing navigation patterns provides opportunities to examine the learning process in multimedia learning environments. This is especially interesting for a specific group that often uses audio-support: learners with dyslexia (Ghesquière et al., 2010; Gregg et al., 2009).

Dyslexia is a learning disability characterized by severe and persistent reading problems that are not due to external factors such as poor education or cognitive problems (Lyon et al., 2003). Even though learners with dyslexia do not have specific difficulties with reading comprehension, their poor decoding skills can hinder them in building a correct text representation and in turn may affect their situation model (Oakhill & Cain, 2017; Snowling, 2013). This can, in turn, negatively affect their reading comprehension (Georgiou et al., 2021).

Audio-support has the potential to reduce learners’ cognitive load due to compensating for reading difficulties. However, audio-support may also overload the working memory and increase external regulation with the risk of reducing reader-initiated decisions. Multimedia research shows that by listening to information, students with dyslexia can compensate for their poor reading skills (Casalis et al., 2013). It is suggested that learning in students with dyslexia could be increased by means of supportive technology to tap into their listening comprehension skills (Fidler &Everatt, 2012). Salmerón and García (2011) showed that higher reading skills predicted a higher ability to strategically adapt learners’ navigation route through multimedial material. In other words, navigation strategies were positively related to reading proficiency (Salmerón & García, 2011; Wu, 2014). In addition, when learners gain more experience in creating mental models of the text, they move from constructing a more or less linear representation — children, towards a more networked based situation model — adults (Klois et al., 2013). Because of their lower reading proficiency, learners with dyslexia could also be disadvantaged regarding navigating through learning environments.

## Navigation strategies and dyslexia

Existing studies do not focus specifically on how learners with dyslexia navigate through learning material. However, research on learning strategies does provide some insights in this regard and shows that these strategies may be different for children and adults. In adults with dyslexia, *larger* amounts of monitoring and time management were reported than in peers without dyslexia (Kirby et al., 2008). These adult learners with learning disabilities used more diverse strategies and preferred additional oral or visual explanations. This is in contrast to the written learning strategies used by typically developing peers (Heiman & Precel, 2003).

Studies on children with dyslexia showed them to have *lower* engagement (more passive learning) as compared to their typically developing peers (Polychroni et al., 2006). It is even posed that children with dyslexia may have lower self-regulation than their typically developing peers in general (Bender & Wall, 1994). However, Bråten and colleagues (2010) showed that well-functioning adolescents with dyslexia use comprehension strategies effectively. In other words, not only do self-regulation skills develop over time (Pintrich & Zusho, 2002), differences between children and adults with dyslexia also seem eminent.

Nevertheless, how exactly audio-support impacts the navigation patterns of learners with dyslexia is far from clear. With regard to gaze behavior, a study in college students found that audio-support affected how learners with dyslexia looked at written text as they focussed less on the written information and made less transitions with audio-support than without, compared to their typically developing peers (Kim & Wiseheart, 2017). Integrating information from different modalities (visual and oral) also turned out to be challenging for them (Kim et al., 2018; MacCullagh et al., 2017). These studies are all focussed on adult learners with dyslexia. Learners with dyslexia seem to process multimedia information differently than typically developing peers, but it is still unclear how audio-support affects self-regulation activities in such environments, and consequentially their navigation patterns. In addition, there is a lack of knowledge on naviagation strategies in young learners with dyslexia.

## The present study

Even though in education audio-support by means of narration is frequently provided to learners, especially learners with dyslexia, the possible impact on learners navigating patterns is unclear. As self-regulation skills develop over time and differences in self-regulation between children and adults with dyslexia have been found, audio-support may impact children’s and adults’ navigation patterns differently. Overall, providing audio-support to learners with and without dyslexia seems to have the risk of impacting their navigation strategies.

Therefore, in the present study, we examined how adding audio affects navigation strategies in children and adults with dyslexia and, in turn, aimed to provide developmental insight into its effect on navigation strategies. In two experimental studies, we compared the navigation strategies of primary school children (experiment 1) and university students (experiment 2) with dyslexia to those of their typically developing peers in multimedia learning environments with and without audio-support.^1^ Navigation strategies were based on the log files of the learning environment. Research questions were:

RQ1 Which navigation patterns can be distinguished in children and adults when learning in a multimedia environment?

RQ2 To what extent does adding audio to multimedia learning environments affect navigation patterns?

RQ3 Does the impact of audio on navigation patterns differ between learners with and without dyslexia?

First, we expected variation in navigation patterns ranging from linear — in which learners linearly go from beginning till end through a lesson — to less linear — in which learners move back and forward between the multimedia slides in various ways. We expected less self-regulation in children than in adults, indicated by a higher number of linear patterns in children.

Secondly, as adding audio-support can be seen as a form of external regulation, we expected that it would decrease reader-initiated decisions. This leads to more linear patterns, especially in adults as children are expected to already show more linear patterns.

Finally, learners with dyslexia were expected to show less self-regulation and more linear navigation patterns than their typically developing peers, especially in adults.

## Experiment one

### Method

#### Participants

A total of 82 grade-5 primary school children were included in the present study, of which 46 typically developing children (70% boys) aged 10.87 years (*SD* = 0.36), and 36 children with dyslexia (64% boys) aged 11.10 years (*SD* = 0.53). All children with dyslexia were diagnosed according to the clinical assessment of the Protocol Dyslexia Diagnosis and Treatment (Blomert, 2005), which assesses children’s reading and a broad range of phonological abilities, inhibition, and memory, and includes environmental factors. Only monolingual children were allowed to participate. Participants were from studies described in Knoop-van Campen et al. (2018, 2019). Some of the children could not be included due to missing log file data as a result of computer malfunction (Knoop-van Campen et al., 2018: 20 children, 50% dyslexia, Knoop-van Campen et al., 2019: 2 children, 0% dyslexia).

Even though all children were in grade 5, the children with dyslexia (*M* = 11.11, *SD* = 0.53) were on average 2 months older than the typical developing children (*M* = 10.87, *SD* = 0.36), *t*(59.43) = 2.372, *p* = 0.021, Cohen’s *d* = 0.53. In line with their diagnosis, children with dyslexia scored significantly lower on word reading^2^ (*M* = 49.39, *SD* = 10.39) and pseudo word reading (*M* = 21.92, *SD* = 6.84) than their typically developing peers (resp. *M* = 71.09, *SD* = 11.04 / *M* = 38.20, *SD* = 9.30), resp. *t*(80) = 9.06, *p* < 0.001, Cohen’s *d* = 2.02 for word reading, *t*(80) = 8.80, *p* < 0.001, Cohen’s *d* = 1.99 for pseudo word reading.

#### Procedure

Testing was done in an individual setting in the schools. All children were provided with two comparable multimedia lessons: pictures with (i) written text, and (ii) written text with audio, offered in a randomized‐block design (one lesson a week). Before the lesson, children were instructed (according to the test protocol) to learn the material as they would get a knowledge test afterwards. It was explained that they could move through the lessons by clicking marked keys on the keyboard. Before the lesson with audio-support, it was explained that they could pause and replay the audio (also with marked keys). In addition, some language tests were performed.

#### Materials

##### Multimedia lessons

The lessons involved biology topics and were chosen from the schoolbooks 1 year above the children’s school year (Van Hoof et al., 2009) to ensure that they had sufficient prior knowledge to understand the material, but at the same time did not receive the information before. The lessons were comparable in set-up and complexity. Each lesson consisted of a title page and 11 content slides (approximately 530 words in total), with every slide showing written text on the left with a supportive picture (Carney & Levin, 2002) on the right. The original paragraphs of the school book text were each placed on a separate slide, thus mimicking the schoolbook with its various text parts on different pages, which contributed to a realistic learning environment (see Knoop-van Campen et al. (2018) for a detailed explanation of the material). Children studied the lessons at their own pace and were able to move back-and-forth through the pages.

##### Audio-support

In one of the two lessons, the material also included audio-support in the form of a voice-over. The voice-over (female voice) read out loud the exact (written) text on a page. The audio started automatically when children clicked to the next slide. Children were able to pause and replay the audio with the keyboard.

##### Log files

Log files of children’s navigation paths through the lessons were recorded by means of timestamps when they moved to a next/previous slide. To identify navigation strategies, all paths were plotted with time (in minutes to increase readability) on the *x*-axis and slide number (1–11) on the *y*-axis (as similar to Jáñez & Rosales, 2016, and Paans et al., 2020).

#### Data-analyses

**Fig. 1:**
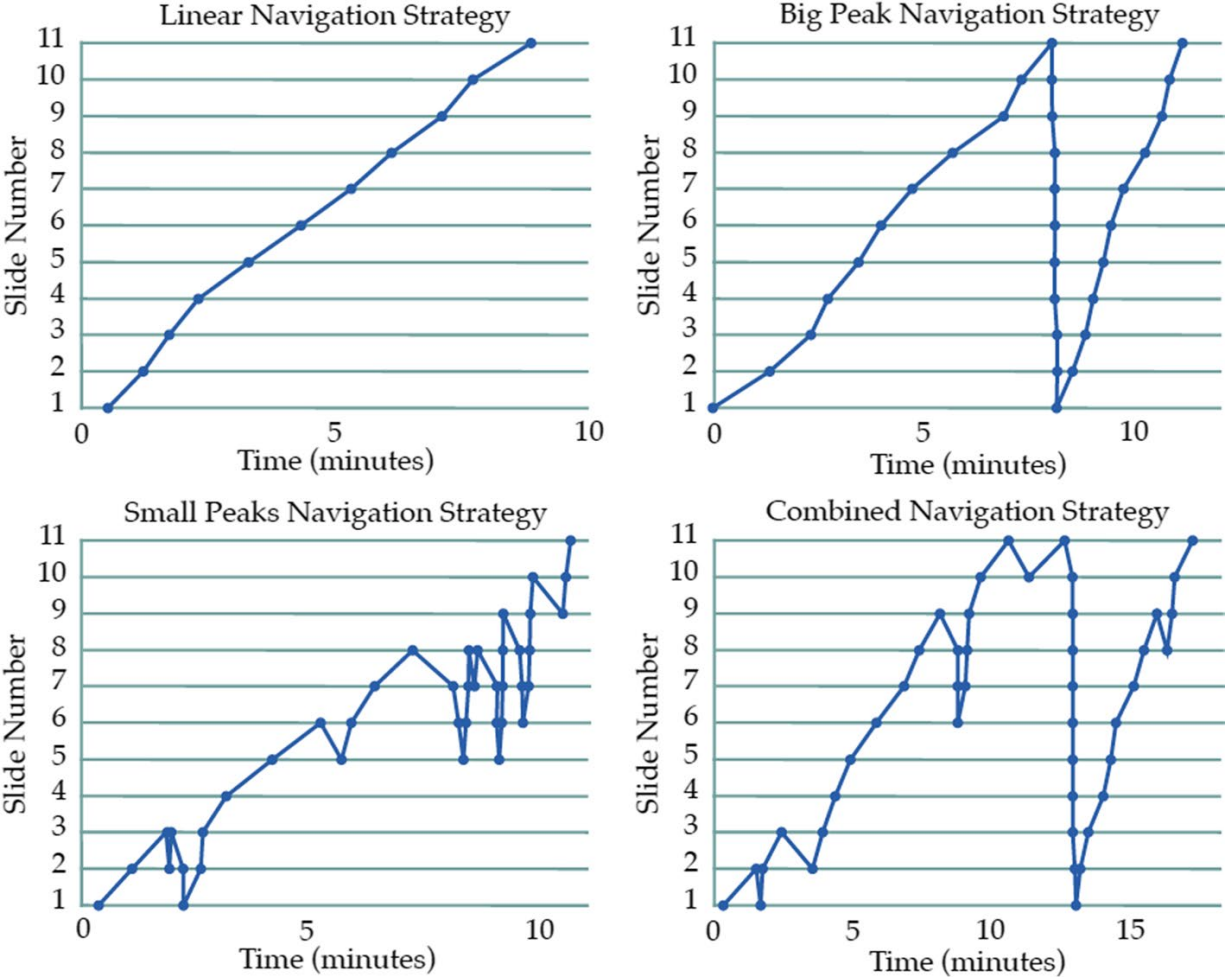
The four navigation patterns

In order to examine the first research question, regarding which navigation patterns could be distinguished, a qualitative analysis of children’s navigation was performed (comparable to Paans et al., 2020). The type of pattern was based on the line graph with time on the *x*-axis and slide number on the *y*-axis for each lesson. The graphs were grouped together based on similarities and differences in their appearance. The graphs with one single line were identified (and thus grouped) first, followed by the graphs with one peak (big peaks). In the remaining graphs, it was noted that all had multiple peaks, but some had one large peak and others had not. The two clusters of small peaks and combined peaks were created. After all patterns were classified, the classification was re-evaluated to see if groups overlapped and could be merged (which was not the case), or if any additional patterns could be derived (which was also not the case). This resulted in a final set of navigation patterns, for which coding criteria were formulated (see Fig. 1 and Table 1). Finally, to check for grouping errors, all graphs were recoded based on this final encoding criteria. To ensure reliability, a second rater rated all the graphs based on the coding criteria. To ensure reliability, a second rater rated all the graphs based on the coding criteria. Inter-rater reliability was good (*κ* = 0.953 (95% *CI*, 0.91 to 1.00), *p* < 0.001).

**Table 1:** Navigation strategies

Navigation strategy	Explanation	Coding
Linear	Participants go through the lesson from the first till the last slide. There is minimal revisiting of previous slides (maximum of one revisit to the preceding slide)	Showing one straight line in the graph
Big peak	Participants go through the lesson linear; however, at the last slide, they go back to the beginning and run through the material a second time revisiting all the pages	Showing one big peak in the graph
Small peaks	When going through the lesson, participants often move back a few slides revisiting part of the slides during learning	Showing multiple small peaks in the graph
Combined peaks	Participants revisiting parts of the slides during learning, but also revisiting all the slides at the end of the lesson	Showing both small peaks and a big peak in the graph

Note. All graphs were be coded in one of the four categories

In order to investigate the second research question, the extent to which adding audio affected the navigation patterns, Wilcoxon tests were used to assess whether there was a significant difference between the two conditions per strategy.

To answer research question three, on whether the impact of audio on navigation patterns differed between children with and without dyslexia, Mann–Whitney tests were used per strategy to determine whether there was a significant difference between the two groups (both conditions combined). Then, to assess whether audio impacted the groups differently (interaction effect), difference scores were calculated for the conditions (0 if the same strategy was used in both conditions, 1 if different strategies were used) and compared between the two groups with additional Mann–Whitney tests.

Due to the small sample sizes, non-parametric analyses were performed. The significance threshold was set to alpha < 0.05, but due to the multiple tests for each strategy, the inflation of alpha error was controlled using the Holm’s step-down procedure (Holm, 1979).

### Results

Four different navigation patterns could be distinguested in primary school children when learning in a multimedia environment children (RQ1), see Fig. 1 and Table 1. Some participants go through the lesson from the first till the last slide (linear). Others do the same but at the last slide, they go back to the beginning and revisit the slides a second time (big peak). Yet others move back and forwards between fewer slides and thus revisit slides during learning (small peaks), and the last group combines navigation between small sets and an extra run through the material (combined peaks). Based on the increase in the number of self-initiated decisions, self-regulation is considered lowest in the linear strategy (children just follow the order of the material) and highest in the combined peaks (children follow their own path regardless of the material).

In primary school children (164 lessons; see Table 2), almost two thirds of the navigation paths were coded as linear, the rest was coded as big, small, or combined peaks.

**Table 2:** Navigation strategies per condition and group

	Text		Text and audio		Total	
	*N*	%	*N*	%	*N*	%
Dyslexia						
Linear	28	78%	25	69%	53	74%
Big peaks	1	3%	5	14%	6	8%
Small peaks	4	11%	4	11%	8	11%
Combined peaks	3	8%	2	6%	5	7%
Typically developing						
Linear	28	61%	28	61%	56	61%
Big peaks	8	17%	6	13%	14	15%
Small peaks	8	17%	11	24%%	19	21%
Combined peaks	2	4	1	2%	3	3%
Total						
Linear	56	68%	53	65%	109	66%
Big peaks	9	11%	11	13%	20	12%
Small peaks	12	15%	15	18%	27	16%
Combined peaks	5	6%	3	4%	8	5%

Results showed no impact of audio on the navigation patterns (RQ2) as there were no significant differences between the text-condition and the text-audio-condition (see Table 3, column “Condition”).

**Table 3:** Main and interaction effects for condition and group, per navigation strategy

	Main effects						Interaction		
	Condition			Group			Condition*Group		
Navigation strategy	*Z*	*p*	*r*	*U*	*p*	*r*	*U*	*p*	*r*
Linear	− 0.63	0.532	0.07	699.00	0.355	0.15	768.00	0.477	0.08
Big peak	− 0.54	0.593	0.06	759.00	0.360	0.10	711.00	0.095	0.18
Small peaks	− 0.73	0.467	0.08	740.00	0.288	0.12	780.00	0.526	0.07
Combined peaks	− 0.82	0.414	0.09	788.50	0.446	0.08	823.00	0.918	0.01

Note. Condition: text vs. text and audio. Group: dyslexia vs. typically developing

Children with and without dyslexia did not differ in the type of navigation strategies and audio did not impact them differently (RQ3), see Table 3, columns “Group” and “Group*Condition.” Children mostly used linear navigation strategies in both conditions and groups.

### Conclusions

In primary school children, four navigation strategies could be observed. In linear patterns, children just follow the order of the material, and thus show little self-regulation. In the other strategies, children increasingly show moments of learners’ self-initiated decisions and increasingly follow their own path regardless of the material.

Primary school children mostly navigate through multimedia environments linearly; adding audio-support to the written text did not change that. As audio-support provides an external prompt and children were already showing a strategy in which they would follow the material, it naturally follows that audio did not impact their navigation strategy.

There were no differences between the use of the four strategies across children with and without dyslexia. Both groups used mostly linear strategies, showing the same amount of self-regulation in their navigation pattern. Audio-support did not impact navigation strategies of primary school children with and without dyslexia.

## Experiment two

### Method

#### Participants

Participants were 85 university and applied-university students. In total, 44 typically developing students (18% men) aged 21.64 years (*SD* = 2.10), and 41 students with dyslexia (15% men) aged 21.78 years (*SD* = 2.42) were included. As in experiment one, students with dyslexia were officially diagnosed with dyslexia. Only monolingual raised students were included. Participants were from the study described in Knoop-van Campen et al. (2020). One participant with dyslexia from that study could not be included due to missing log file data.

The university students with and without dyslexia did not differ in age, *t*(83) = 0.29, *p* = 0.770, Cohen’s *d* = 0.06. In line with their diagnosis and despite their educational level, students with dyslexia scored significantly lower on word reading^3^ (*M* = 79.76, *SD* = 11.38) and pseudo word reading (*M* = 71.39, *SD* = 18.64) than typically developing students (resp. *M* = 95.77, *SD* = 14.63 / *M* = 96.63, *SD* = 13.26), resp. *t*(83) = 5.61, *p* < 0.001, Cohen’s *d* = 1.22 for word reading, *t*(83) = 7.32, *p* < 0.001, Cohen’s *d* = 1.56 for pseudo word reading.

#### Procedure

Similar as in experiment one, testing was done in an individual setting and all students were provided with two comparable multimedia lessons: pictures with (i) written text, and (ii) written text with audio, offered in a randomized‐block design (one lesson a week). Students received similar instructions before the lesson as in experiment one regarding the purpose of the lesson (to learn for a knowledge test) and the audio-support. Also, similar language tests were performed.

#### Materials

##### Multimedia lessons

As in experiment one, the lessons involved biology topics but then based on the curriculum of the first study year of biology at university level (Campbell Biology: Reece et al., 2014). One lesson consisted of a title page and 15 content slides (900 words in total), with every slide showing written text with a picture. Students studied the lessons at their own pace and were able to move back-and-forth through the pages.

##### Audio-support

Audio-support was similar to experiment one. The voice-over (female voice) read out loud the exact (written) text on a page. The audio started automatically and could be paused and replayed.

##### Log files

Log file recording and coding was similar to experiment one. Like in experiment one, inter-reliability was good (*κ* = 0.953 (95% *CI*, 0.84 to 0.95), *p* < 0.001).

#### Data-analyses

Analyses were similar to experiment one.

### Results

The same four navigation strategies as in experiment one could be distinguished: linear, big peaks, small peaks, and combined peaks (RQ1), see Fig. 1 and Table 1.

In university students (170 lessons; see Table 4), over one third of the navigation paths were coded as combined peaks. Linear and small peaks were each coded a quarter of the lessons, and big peaks were coded the least.

**Table 4:** Navigation strategies per group and condition

	Text		Text and audio		Total	
	*N*	%	*N*	%	*N*	%
Dyslexia						
Linear	7	17%	12	29%	19	23%
Big peaks	4	10%	9	22%	13	16%
Small peaks	13	32%	7	17%	20	24%
Combined peaks	17	41%	13	32%	30	37%
Typically developing						
Linear	8	18%	13	30%	21	24%
Big peaks	7	16%	6	14%	13	15%
Small peaks	9	20%	12	27%	20	24%
Combined peaks	20	45%	13	30%	34	38%
Total						
Linear	15	18%	25	29%	40	24%
Big peaks	11	13%	15	18%	26	15%
Small peaks	22	26%	19	22%	40	24%
Combined peaks	37	44%	26	31%	64	37%

Results showed that audio impacted the type of navigation patterns (RQ2) as there were significant differences between the text-condition and the text-audio-condition for linear and combined strategies: more linear and less combined strategies were used in the text-audio-condition than in the text-condition (see Table 5, column “Condition”).

Students with and without dyslexia did not differ in the type of navigation strategies and audio impacted them similarly (RQ3), see Table 5, columns “Group” and “Group*Condition.”

**Table 5:** Main and interactions effects for condition and group, per navigation strategy

	Main effects						Interaction		
	Condition			Group			Condition*Group		
Navigation strategy	*Z*	*p*	*r*	*U*	*p*	*r*	*U*	*p*	*r*
Linear	− 2.50	**0.012**	0.27	886.50	0.870	0.02	897.00	0.948	0.01
Big peak	− 1.00	0.317	0.11	871.50	0.722	0.04	782.50	0.123	0.17
Small peaks	− 0.63	0.532	0.07	886.00	0.870	0.02	734.00	0.058	0.21
Combined peaks	− 2.40	**0.016**	0.26	891.00	0.916	0.01	853.00	0.567	0.06

Bold are the significant results (sig. *p*-values)

Note. Condition: text vs. text and audio. Group: dyslexia vs. typically developing

### Conclusions

Like in experiment one, four navigation strategies could be distinguished, which increase in the number of learners’ self-initiated decisions and whether they follow their own path regardless of the material. Audio-support changes navigation strategies in adults towards a strategy reflecting less self-regulation and does so similarly for adults with and without dyslexia.

## General discussion

In the present study, we examined how children and adults with and without dyslexia navigate through multimedia learning environments and aimed to provide insight into the developmental perspective of navigation strategies. In two experiments, it was examined to what extent adding audio-support to written text impacted the navigation strategies in multimedia lessons of primary school children and university students with dyslexia as compared to those of their typically developing peers. Log files were recorded to identify the strategies. Children showed mostly linear navigation strategies in both conditions. Adults used mostly combined peaks strategies in the text-condition, but with audio-support, adults used more linear and less combined strategies. In neither group, differences were found between learners with dyslexia and the controls.

### Navigation strategies in multimedia learning

In line with the first hypothesis, we found several navigation strategies including one clear linear navigation strategy. In this linear navigation strategy, learners did not revisit previous pages during the lessons or at the end of the lesson. The other three strategies — linear reading of the whole chapter after which the chapter is reread once (big peak), reading with often going back to previous pages during the lesson (small peaks), and a combination of at least one big and multiple small peaks strategies (combined peaks) — show increased moments of learners’ self-initiated decisions during learning in the multimedia environment. The navigation paths show an increase in deliberate actions through the material in a way that suggest that learners are actively involved in their learning process, which is expected to foster their comprehension and learning outcomes (Zimmermann, 2000; Van den Broek & Helder, 2017). As Greene and Azevedo (2010) put forward in the introduction of their special issue on the measurement of self-regulation in computer-assisted learning environments, sequential and temporal patterns as shown in navigation patterns can provide insight into learners’ self-regulation behavior (Greene & Azevedo, 2010; Saint et al., 2020). Navigation patterns may thus be interpreted as a proxy for (the amount of) self-regulation (Saint et al., 2020).

Children were found to show mainly linear navigation strategies, as could be expected based on their less developed self-regulation skills (De Jong & van Joolingen, 1998). Going in a linear fashion through the multimedia environment could of course also have been the most expeditious strategy. If the material was precisely adjusted to the student’s proficiency level, there seems to be little reason — especially for a student with relatively little self-regulation — to intensively engage with the material. Had the content been harder, maybe students would have shown other patterns than merely the linear one. Adults with more developed regulation skills (Zimmerman, 2000) and more experience in navigating through multimedia environments (Mead et al., 1997) showed, as expected, more self-initiated decisions (reflecting self-regulation) by means of revisiting previous pages.

Mostly in line with the second hypothesis, audio-support impacted navigation strategies but only in adults and not in primary school children. Children in this study navigated through the multimedia environments linearly and generally did not revisit previous pages; the additional audio did not change that. As audio-support provided an external prompt and children were already showing a strategy in which they followed the material, it is no surprise that the audio did not impact their navigation strategies. As the adults used navigation strategies that reflected more self-regulation, they showed more revisiting of previous pages. In turn, the audio affected their navigation strategies towards a strategy reflecting less self-regulation, as with audio, they showed less combined peak strategies and more linear strategies. With audio-support, they were less likely to revisit previous information. Pintrich and Zusho (2002) explained that regulation skills develop not only as a function of age but also of experience with the specific task (in this case, learning in multimedia environments). Our results show that audio-support can indeed be considered an external prompt that changes learning behavior in highly educated and experienced learners.

This has the important implication for the CTML that the impact of audio seems to differ between learners. The present results, combined with Knoop-van Campen et al. (2022) who showed that audio support has a negative effect on students’ reading comprehension strategy, indicate that information processing is active and that audio can reduce the number of reader-initiated actions: audio seem to make learning less active. For optimal learning, a learner must actively engage in the material (Caccamise et al, 2015). It appears that audio support takes over part of the control and learners consequently become less active with the content. This may lead to lower immediate and long-term learning gains.

Theoretically, according to the CTML, the idea of the redundancy principle leans on (over)loading the cognitive capacity (working memory) of a learner. Out results indicate that the redundancy principle may also depend on learners’ self-regulation capacities and the extent to which learners actively engage in the material. This naturally points towards differences between children and adults, as demonstrated in the present study, and the need for a developmental perspective on (certain aspects of) multimedia learning.

### Navigation strategies and dyslexia

Differences were also expected between learners with and without dyslexia (hypothesis 3); however, none were found. This may be explaind by the fact that despite their decoding problems, learners with dyslexia officially do not have specific comprehension problems (Lyon et al., 2003). The studies that show relations between reading skills and navigation strategies used measures focused on reading comprehension, rather than on technical reading skills (Salmerón & García, 2011; Wu, 2014). Next to this, students with dyslexia — in contrast to poor comprehenders — use context to compensate for their reading problems (Nation & Snowling, 1998), which implies the use of certain reading strategies. Bråten and colleagues (2010) indeed showed that students with dyslexia can use comprehension strategies efficiently and use different coping strategies to compenstate for their decoding problems.

An important difference with the studies that did find differences between students with and without dyslexia on (academic learning) strategies is that they measured strategy use by means of self-questionnaires (Heman & Parcel, 2003; Kirby, et al., 2008; Polychroni et al., 2006). Such measures rely heavily on learners’ memory and/or reflective capacities (Paans et al., 2020) and may, therefore, be less reliable. These questionnaires furthermore inquire more about generic study techniques, whereas in the current study, we investigated learning behavior in specific multimedia lessons. A study, in which the eye-movements of secondary school students with and without dyslexia were compared during a reading comprehension task, showed no differences in the reading strategy both groups used (Knoop-van Campen et al., 2022).

In addition, learners with dyslexia generally read slower, but as our navigation strategies were coded based on the visual display of the navigation strategies, reading time was not considered. This allowed us to purely examine their navigation pattern and shows that even though there are differences between learners’ with and without dyslexia on micro-level (e.g., reading skills, and see also Kim & Wiseheart, 2017), their navigation strategies to tackle a learning task are comparable. In line with suggestions from Knoop-van Campen et al. (2022), a more time-restricted assignment may have led to differences between learners with and without dyslexia. Time pressure taps into their decoding problems and increases cognitive load, which could have reduced their capacity to make self-initiated decisions.

Another explanation why we did not find differences between learners with and without dyslexia could be that audio-support may both foster and hamper learners with dyslexia. On the one hand, audio has the potential to facilitate text representation but, on the other hand, it may also hinder learners’ active involvement. It might be the case that these two aspects co-occur and cancel each other out on the navigation paths. This way, no differences would arise even though the mechanism behind navigation could be different for learners with and without dyslexia.

### Limitations and future research

Some limitations can be put forward. First of all, the fact that audio-support affects navigation strategies in adults directly raises questions about its impact on students’ learning outcome. Unfortunately, the measures in these studies are not suitable for analyzing this relation due to the randomized-block design, which complicates the interpretation of learning outcome results in combination with low numbers of some of the navigation strategies. In addition, the knowledge questions used in the two primary school studies (Knoop-van Campen et al., 2018, 2019) were not the same. Future research may investigate the link between navigation strategies and learning outcomes by adapting the set-up to suit this aim. As differences in learners’ navigational behavior was found to be related to their cognitive learning styles (Graf & Liu, 2010), future research could also investigate how the effect of navigation strategies on learning outcomes may depend on learning styles.

Second, it would be interesting to measure learners’ self-regulation skills, to be able to validate the interpretation of the plotted graphs. However, as the results are clearly focused on the two most distant navigation strategies (linear and combined peaks), this validation is not likely to change the interpretation of the explained studies.

Third, while the first and fourth navigation strategies (linear and combined peaks) can be clearly defined in terms of regulation activities, one could debate which of the other two patterns (big peak / small peaks) reflects more self-regulation. We chose to order big peaks as “less regulation,” as learners with small peaks show more regulation decisions (instead of only once at the end of the lesson).

Finally, it is demonstrated that the exact navigation strategies are highly dependent on system characteristics. In a linear multimedia scenario, which was used in the present study, participants are only able to go back and forth through the slides. In the absence of hyperlinks within the text, possibilities to actively navigate the system are limited compared to, for example, hypermedia and learning on the internet — a typical learning environment in which navigation is important. To generalize the results of the present study to other environments, future research on navigation paths could use a more hierarchical or network-based learning environment. It should be noted that the linear set-up of the multimedia lessons was also a positive feature, as the effect of narration could be investigated. As learners with dyslexia often use software that reads the written text out loud to them when they read their schoolbooks on their computer screens, our results close a tap between “typical” learning from a paper school book and learning in a hypermedia setting. In a similar vein, it could be worthwhile and of practical relevance to examine the extent to which these results on nonfiction can be extrapolated to fictional texts, such as books for reading.

### Practical implications

There is an urgent need to understand how audio-support affects navigation strategies in learners with dyslexia. As the development of navigation strategies in learners with dyslexia is unidentified, less adequate counseling can be given regarding the use of audio-support in education. Since navigation strategies are important for learning, understanding the impact of individual differences on these navigation strategies is important. Many educators and educational designers use audio to support readers with dyslexia and these practitioners need information on how implementation of multimedia affects students’ learning behavior. This study adds to existing multimedia learning knowledge in such a way that it transcends purely learning outcomes, while focusing on what happens *during* learning and how this develops. This developmental perspective is an uncultivated research area within multimedia learning.

The present study shows that the compensational components in education — which is often audio-support in the form of narrating the written text (Ghesquière et al., 2010) — have a different impact on young children than on adults. It raises the question where the tipping point is for audio to start affecting learning behavior. This might already be the case at secondary education as is shown in Knoop-van Campen et al. (2022) where secondary school students approach reading comprehension tasks less efficiently with audio than without audio (longer time, less efficient strategy). We know that in adults with and without dyslexia, audio support impacts learning outcomes negatively (Knoop-van Campen et al., 2020). We therefore advocate that costs and benefits of audio-support should be carefully considered per learner, as it can affect how (adult) students learn. By interacting with a learner and observing work accomplished with and without audio support, practitioners can identify — together with the (adult) learner — the best approach for this person. Schraw’s (2007) called for an understanding of the impact of regulation process on learning in computer-based learning environments. In a similar vein, we argue that raising awareness and providing instruction about navigation strategies with its possible impact of audio is important for learners, in order to optimally work with audio support. Just like they need guidance to learn from text and use appropriate strategies, they also need instruction and support on how to learn with digital aids (see also Scheltinga & Siekman, 2020).

### Conclusions

In the present paper, we showed that audio-support changes navigation strategies but only for adults, and that it does so similarly for adult learners with and without dyslexia. Whereas children tend to navigate linearly through multimedia learning environments, adult learners use diverse navigation strategies, which tend to reflect less self-regulation in the case of audio-support. This implicates that for adults, audio-support may be less desirable when the goal is to learn the material. It also emphasizes the need for further research on the effects of navigation strategies on learning outcomes.

## Footnotes

1 Log file data (a.k.a. time stamps of the lesson slides) were taken from three experimental studies on multimedia learning and dyslexia (Knoop-van Campen et al., 2018, 2019, 2020). This log file data was not previously used for analysis or publication elsewhere and the data is unique for this study.

2 To measure (pseudo) word decoding, the Een-Minuut-Test (EMT) [One-Minute-Test] (Brus & Voeten, 1999) and the Klepel (Van den Bos, Spelberg, Scheepsma, & De Vries, 1994) were used. In both tests, learners had to read as many (pseudo) words as possible within 1 min. Total score is the number of words read correctly.

3 The same (pseudo) word decoding tests were used as in experiment 1. Total score is the number of words read correctly in 1 min (EMT) and 2 min (Klepel).

## References

[CR1] Azevedo R, Cromley JG (2004). Does training on self-regulated learning facilitate students’ learning with hypermedia?. Journal of Educational Psychology.

[CR2] Barab SA, Fajen BR, Kulikowich JM, Young MF (1996). Assessing hypermedia navigation through Pathfinder: Prospects and limitations. Journal of Educational Computing Research.

[CR3] Bender WN, Wall ME (1994). Social-emotional development of students with learning disabilities. Learning Disability Quarterly.

[CR4] Blomert, L. (2005). *Dyslexie in Nederland*. Uitgeverij Nieuwezijds.

[CR5] Boekaerts M, Corno L (2005). Self-regulation in the classroom: A perspective on assessment and intervention. Applied Psychology.

[CR6] Bråten I, Amundsen A, Samuelstuen MS (2010). Poor readers-good learners: A study of dyslexic readers learning with and without text. Reading & Writing Quarterly.

[CR7] Brus BT, Voeten MJM (1999). Een-minuut-test: Vorm A en B: Verantwoording en handleiding: Schoolvorderingentest voor de technische leesvaardigheid, bestemd voor groep 4 tot en met 8 van het basisonderwijs.

[CR8] Caccamise, D., Friend, A., Littrell-Baez, M. K., & Kintsch, E. (2015). Constructive theory as a framework for instruction and assessment of reading. In S. R. Parris & K. Headley (Eds.), *Comprehension instruction: Research-based best practices* (3rd ed., pp. 88–104). Guilford Press.

[CR9] Carney RN, Levin JR (2002). Pictorial illustrations still improve students’ learning from text. Educational Psychology Review.

[CR10] De Jong T, van Joolingen WR (1998). Scientific discovery learning with computer simulations of conceptual domains. Review of Educational Research.

[CR11] Georgiou, G. K., Martinez, D., Vieira, A. P. A., Antoniuk, A., Romero, S., & Guo, K. (2021). A meta-analytic review of comprehension deficits in students with dyslexia. *Annals of Dyslexia,* 1–45. 10.1007/s11881-021-00244-y 34532777

[CR12] Ghesquière P, Boets B, Gadeyne E, Vandewalle E, Geudens A, Baeyens D, Schraeyen K, Maetens K, De Brauwer J, Loncke M (2010). Dyslexie: Een beknopt wetenschappelijk overzicht. Jongvolwassenen met dyslexie: Diagnostiek en begeleiding in wetenschap en praktijk.

[CR13] Graesser AC, McNamara DS (2007). An introduction to strategic reading comprehension. Reading comprehension strategies: Theories, interventions, and technologies.

[CR14] Graf S, Liu TC (2010). Analysis of learners’ navigational behaviour and their learning styles in an online course. Journal of Computer Assisted Learning.

[CR15] Greene JA, Azevedo R (2010). The measurement of learners’ self-regulated cognitive and metacognitive processes while using computer-based learning environments. Educational Psychologist.

[CR16] Gregg N, Banerjee M, Reid G (2009). Reading comprehension solutions for college students with dyslexia in an era of technology. Dyslexia: A handbook for research and practice.

[CR17] Heiman T, Precel K (2003). Students with learning disabilities in higher education: Academic strategies profile. Journal of Learning Disabilities.

[CR18] Holm S (1979). A simple sequentially rejective multiple test procedure. Scandinavian Journal of Statistics..

[CR19] Jáñez Á, Rosales J (2016). Novices’ need for exploration: Effects of goal specificity on hypertext navigation and comprehension. Computers in Human Behavior.

[CR20] Jeske D, Backhaus J, Stamov Roßnagel C (2014). Self-regulation during e-learning: Using behavioural evidence from navigation log files. Journal of Computer Assisted Learning.

[CR21] Juvina I, Van Oostendorp H (2008). Modeling semantic and structural knowledge in web navigation. Discourse Processes.

[CR22] Kim S, Wiseheart R (2017). Exploring text and icon graph interpretation in students with dyslexia: An eye-tracking study. Dyslexia.

[CR23] Kim S, Wiseheart R, Walden PR (2018). Do multimedia instructional designs enhance comprehension in college students with dyslexia?. Journal of Postsecondary Education and Disability.

[CR24] Kirby JR, Silvestri R, Allingham BH, Parrila R, La Fave CB (2008). Learning strategies and study approaches of postsecondary students with dyslexia. Journal of Learning Disabilities.

[CR25] Knoop-van Campen, C. A. N., Segers, E., & Verhoeven, L. (2018). The modality and redundancy effects in multimedia learning in children with dyslexia. *Dyslexia, 24*(2), 140–155. 10.1002/dys.1585 10.1002/dys.1585 PMC6084336 29577504

[CR26] Knoop-van Campen, C. A. N., Segers, E., & Verhoeven, L. (2019). Modality and redundancy effects, and their relation to executive functioning in children with dyslexia. *Research in Developmental Disabilities, 90*, 41–50. 10.1016/j.ridd.2019.04.007 10.1016/j.ridd.2019.04.007 31051311

[CR27] Knoop-van Campen, C. A. N., Segers, E., & Verhoeven, L. (2020). Effects of audio support on multimedia learning processes and outcomes in students with dyslexia. *Computers & Education, 150*, 103858. 10.1016/j.compedu.2020.103858

[CR28] Knoop-van Campen, C. A. N., Ter Doest, D., Verhoeven, L., & Segers, E. (2022). The effect of audio-support on strategy, time, and performance on reading comprehension in secondary school students with dyslexia. *Annals of Dyslexia, 72*(2), 341–360. 10.1007/s11881-021-00246-w 10.1007/s11881-021-00246-w PMC9187546 34797513

[CR29] Lawless KA, Kulikowich JM (1996). Understanding hypertext navigation through cluster analysis. Journal of Educational Computing Research.

[CR30] Lyon GR, Shaywitz SE, Shaywitz BA (2003). A definition of dyslexia. Annals of Dyslexia.

[CR31] MacCullagh L, Bosanquet A, Badcock NA (2017). University students with dyslexia: A qualitative exploratory study of learning practices, challenges and strategies. Dyslexia.

[CR32] Mayer RE (2005). The Cambridge Handbook of Multimedia Learning. Cambridge University Press.

[CR33] Mayer RE, Fiorella L, Mayer RE (2014). 12 principles for reducing extraneous processing in multimedia learning: Coherence, signaling, redundancy, spatial contiguity, and temporal contiguity principles. The Cambridge handbook of multimedia learning.

[CR34] Mead SE, Spaulding VA, Sit RA, Meyer B, Walker N (1997). Effects of age and training on World Wide Web navigation strategies. Proceedings of the human factors and ergonomics society annual meeting.

[CR35] Nation K, Snowling MJ (1998). Individual differences in contextual facilitation: Evidence from dyslexia and poor reading comprehension. Child Development.

[CR36] Paans C, Molenaar I, Segers E, Verhoeven L (2020). Children’s macro-level navigation patterns in hypermedia and their relation with task structure and learning outcomes. Frontline Learning Research.

[CR37] Pieschl S, Stahl E, Murray T, Bromme R (2012). Is adaptation to task complexity really beneficial for performance?. Learning and Instruction.

[CR38] Pintrich PR, Zusho A, Wigfield A, Eccles JS (2002). The development of academic self-regulation: The role of cognitive and motivational factors. Development of achievement motivation.

[CR39] Polychroni F, Koukoura K, Anagnostou I (2006). Academic self-concept, reading attitudes and approaches to learning of children with dyslexia: Do they differ from their peers?. European Journal of Special Needs Education.

[CR40] Reece, J. B., Urry, L. A., Cain, M. L., Wasserman, S. A., Minorsky, P. V., & Jackson, R. B. (2014). *Campbell biology* (p. 135). Pearson.

[CR41] Saint, J., Gašević, D., Matcha, W., Uzir, N. A. A., & Pardo, A. (2020). Combining analytic methods to unlock sequential and temporal patterns of self-regulated learning. *Proceedings of the Tenth International Conference on Learning Analytics & Knowledge*, 402-411. 10.1145/3375462.3375487

[CR42] Salmerón L, García V (2011). Reading skills and children’s navigation strategies in hypertext. Computers in Human Behavior.

[CR43] Scheltinga, F. & Siekman, B. (2020, 12 November). Literatuurstudie naar de effectiviteit van technische hulpmiddelen bij dyslexie. Geraadpleegd van https://dyslexiecentraal.nl/sites/default/files/media/ document/2021–07/20210701%20Review%20Hulpmiddelen%20Dyslexie%20Centraal.pdf

[CR44] Schraw G (2007). The use of computer-based environments for understanding and improving self-regulation. Metacognition and Learning.

[CR45] Van den Bos, K. P., Spelberg, H., Scheepsma, A., & De Vries, J. (1994). De Klepel. Vorm A en B. Een test voor de leesvaardigheid van pseudowoorden. Verantwoording, handleiding, diagnostiek enbBehandeling. *Berkhout*.

[CR46] Van den Broek P, Helder A (2017). Cognitive processes in discourse comprehension: Passive processes, reader-initiated processes, and evolving mental representations. Discourse Processes.

[CR47] Van Hoof, K., Siemensma, F., Smit, J., & Vegh, G. (2009). *Wijzer door natuur & techniek.* Noordhoff Uitgevers.

[CR48] Winne PH, Hadwin AF, Hacker DJ, Dunlosky J, Graesser AC (1998). Studying as self-regulated learning. Metacognition in educational theory and practice.

[CR49] Wu JY (2014). Gender differences in online reading engagement, metacognitive strategies, navigation skills and reading literacy. Journal of Computer Assisted Learning.

[CR50] Zimmerman B, Boekaerts M, Pintrich P, Zeidner M (2000). Attaining self-regulation: A social cognitive perspective. Handbook of self–regulation.

